# Cellular immune responses in patients with hepatitis B surface antigen seroclearance induced by antiviral therapy

**DOI:** 10.1186/1743-422X-8-69

**Published:** 2011-02-14

**Authors:** Minfeng Liang, Shiwu Ma, Xiaoxiong Hu, Bin Zhou, Junchang Zhang, Jinjun Chen, Zhanhui Wang, Jian Sun, Xiaolin Zhu, William Abbott, Jinlin Hou

**Affiliations:** 1 Hepatology Unit and Key Lab for Organ Failure Research, Nanfang Hospital, Southern Medical University, Guangzhou 510515, P.R. China; 2 Department of Liver Diseases, Zhuhai Branch Guangdong Provincial Hospital of Traditional Chinese Medicine, Zhuhai 519015, P.R. China; 3 New Zealand Liver Transplant Unit, Auckland Hospital, Private Bag 92-024, Auckland, New Zealand

## Abstract

**Background:**

The mechanisms by which chronic hepatitis B is completely resolved through antiviral therapy are unknown, and the contribution of acquired T cell immunity to hepatitis B surface antigen (HBsAg) seroclearance has not been investigated. Therefore, we measured the T-cell responses to core and envelope antigens in patients with HBsAg seroclearance.

**Methods:**

Fourteen subjects with HBsAg seroclearance following antiviral treatment for chronic hepatitis B, 7 HBeAg-positive immunotolerant HBV carriers and 9 HBeAg-negative inactive HBsAg carriers were recruited. HBV-specific T-cell responses to recombinant HBV core (rHBcAg) and envelope (rHBsAg) proteins and pools of core and envelope peptides were measured using an ELISPOT assay detecting interferon-gamma and intracellular cytokine staining (ICS) assays detecting interferon-gamma or interleukin 2.

**Results:**

Interferon-gamma ELISPOT assays showed a low frequency of weak responses to the rHBsAg and S peptide pool in the HBsAg seroclearance group, and the response frequency to the rHBcAg and the C peptide pool was higher than to the rHBsAg (*P *< 0.001) and S peptide pool (*P *= 0.001) respectively. A higher response frequency to C than S peptide pools was confirmed in the interferon-gamma ICS assays for both CD4+ (*P *= 0.033) and CD8+ (*P *= 0.040) T cells in the HBsAg seroclearance group. The responses to C and S antigens in the inactive carriers were similar.

**Conclusions:**

There was a low frequency of CD4+ and CD8+ T cell immune responses to envelope antigens in Chinese subjects with HBsAg seroclearance following antiviral therapy. It is unlikely that these immune responses are responsible for HBsAg seroclearance in these subjects.

## Background

Major advances have been made in the treatment of hepatitis B during the past 10 years. However, much remains to be accomplished because current antiviral therapy does not permanently eradicate infection. A permanent suppression of HBV replication following treatment will require generation of a robust acquired immune response against peptides contained within the HBV core and envelope genes [1]. There is some evidence that drug-induced suppression of the HBV results in increased acquired immunity against the virus [2-4]; possibly because the decrease in the levels of core and particularly envelope antigens allows T cells to recover from exhaustion [5]. The possibility that this may be an important mechanism leading to complete recovery from chronic hepatitis B has not yet been explored in vivo.

A small number of patients taking modern antiviral therapies achieve a complete loss of both core and envelope proteins from serum. This process is known as HBsAg seroclearance. It would be of interest to know if there was an increase in acquired immunity to envelope antigens in these subjects, as this would allow two hypotheses to be tested. The first hypothesis is that the acquired immune response to the envelope protein contributes to HBsAg seroclearance. The second hypothesis is that the decrease in serum HBsAg levels would reverse the exhaustion of CD8+ T cells that respond to envelope peptides [5]. In this study, we have investigated the T-cell responses to peptides encoded by the HBV C and S open reading frames in patients with HBsAg seroclearance following antiviral therapy.

## Methods

### Patients

A total of 30 patients were enrolled in the study. They were recruited from the Hepatology Unit of Nanfang Hospital in Southern Medical University, and the Department of Liver Diseases in Zhuhai Branch Guangdong Provincial Hospital of Traditional Chinese Medicine. There were 14 patients with sustained HBsAg seroclearance (HBsAg < 0.05 IU/mL) following treatment of their HBeAg-positive chronic hepatitis B with either pegylated interferon α (n = 3), nucleot(s)ide analogue treatment (n = 8) or sequential treatement with pegylated interferon α and nucleot(s)ide analogue (n = 3). They were off-treatment when the blood was taken, and all subjects had an ALT of < = 60 IU/L. At baseline, they were all positive for HBeAg, had an HBV DNA level > 5.0 log_10 _copies/mL and a serum ALT > 80 IU/L. The controls were 9 inactive HBsAg carriers (HBsAg positive, anti-HBe positive, anti-HBc positive, ALT < 40 IU/L, HBV DNA < 3.0 log_10 _copies/mL; representing the inactive hepatitis B phase) and 7 immunotolerant HBV carriers (HBsAg positive, HBeAg positive, anti-HBc positive, ALT < 40 IU/L, HBV DNA > 4.0 log_10 _copies/mL; representing the immune tolerant phase). The presence of HCV/HIV infection, hepatocellular carcinoma, autoimmune liver disease, other malignant tumors and severe metabolic diseases was excluded in all patients. The clinical characteristics of these patients are summarized in Table 1 and Table 2. In this investigation, we enrolled the patients with normal ALT level in sera (only one patient in HBsAg seroclearance group with ALT at 60 IU/L), in order to ensure that all subjects were under homogeneity of hepatic inflammation.

**Table 1:** The HBV seromarkers, genotypes and mutations in patients with HBsAg seroclearance

Patient	Genotype	Drugs	HBsAg^a^	HBsAb^a^	HBeAg^a^	HBeAb^a^	HBcAb^a^	Mutations in RT	Mutations in S
SC-1	UD	ETV	NEG	NEG	NEG	NEG	POS	UD	UD
SC-2	UD	LAM	NEG	NEG	NEG	POS	POS	UD	UD
SC-3	UD	ETV	NEG	POS	POS^b^	NEG	POS	UD	UD
SC-4	UD	LAM	NEG	POS	NEG	POS	POS	UD	UD
SC-5	B	Peg-IFN	NEG	NEG	NEG	POS	POS	L199V	T47A, C48R, S61L, I68T, L109P, W196L, I208T
SC-6	C	Peg-IFN, ETV	NEG	POS	POS^b^	NEG	POS	T54S, H55R P109Q	T47A, I68T Q101K, P111S, T123K, I126T, P203R
SC-7	C	LAM	NEG	NEG	NEG	NEG	POS	H55R, V56A; L80I, L180M, M204I	T47A, C48R, S61L, I68T, L109P, W196L
SC-8	UD	Peg-IFN, LAM	NEG	POS	NEG	POS	POS	UD	UD
SC-9	UD	Peg-IFN	NEG	POS	NEG	NEG	POS	UD	UD
SC-10	C	Peg-IFN, LAM	NEG	POS	NEG	NEG	POS	S53T, H55S Y124H, M129L	A45L, T47V, W199L, Y200F
SC-11	C	LAM	NEG	NEG	NEG	NEG	POS	Y124H, V207I	M198L, W199L, Y200F
SC-12	UD	Peg-IFN	NEG	POS	NEG	NEG	POS	UD	UD
SC-13	C	LAM	NEG	NEG	NEG	NEG	POS	H55R, V56A L80I, L180M, M204I	T47A, C48R, S61L, I68T L109P, W196L
SC-14	UD	LAM	NEG	POS	NEG	NEG	POS	UD	UD

Note. SC, patient with HBsAg seroclearance; UD, HBV DNA undetectable by nested PCR; ETV, entecavir; LAM, lamivudine; Peg-IFN, pegylated IFN; NEG, negative; POS, positive; RT, reverse transcriptase; S, S open reading frame; HBsAb, antibody to HBsAg; HBeAb, antibody to HBeAg; HBcAb, antibody to HBcAg.

^a ^HBV serum markers were all determined by chemiluminescent microparticle immunoassay through Abbott ARCHITECT^® ^i2000 system.

^b ^Two of these were seropositive for HBeAg at the time we collected the blood sample. However, both subjects became HBeAg-negative during follow-up.

**Table 2:** The demographic and clinical features of all subjects in this investigation

	Subjects			P value
	
	HBsAg seroclearance (n = 14)	Immunotolerant HBV carrier (n = 7)	Inactive HBsAg carrier (n = 9)	

Age(yrs) Median/Range	38/21~58	23/20~36	25/22~34	0.002^a^
Gender Male/Female	11/3	5/2	5/4	0.499^b^
ALT(U/L) Median/Range	22/13~60	33/20~39	21/9~39	0.144^a^
HBV DNA (Log_10 _copies/mL) Mean ± SD	undetectable^c^	7.05 ± 0.66	undetectable^c^	

Note. **^a ^**Kruskal-Wallis H test; ^b ^Fisher's exact test; ^c ^HBV DNA undetectable by real-time PCR

The quantitation of HBsAg in patients with HBsAg seroclearance was determined by the Abbott Architect^® ^i2000 System, and the ARCHITECT HBsAg assay was obtained from Abbott Ireland Diagnostics Division, which was a chemiluminescent microparticle immunoassay for quantitative determination of HBsAg in human serum and plasma. The ARCHITECT HBsAg assay has a sensitivity of ≤ 0.05 IU/mL. The most prevalent HBsAg mutants, such as the G145A mutant, are readily detected in the ARCHITECT HBsAg assay with a sensitivity equivalent to detection of wild type HBsAg.

The study was conducted according to the guidelines of the Declaration of Helsinki, and was approved by the Ethical Committee of Nanfang hospital in Southern Medical University. All subjects gave informed consent.

### HBV Genotyping in Patients with HBsAg Seroclearance

HBV genotypes in patients with HBsAg seroclearance were assigned as described previously [6]. Briefly, HBV DNA was extracted from serum samples with the QIAGEN MinElute Viral Spin kit (QIAGEN, Germany) following the manufacturer's recommendations. Amplimers covering AA46-217 of the P open reading frame or AA38-208 of the S open reading frame were amplified. Sequence alignments were performed using ClustalX v1.8. Phylogenetic tree analysis was performed using the Tamura-Nei model of evolutionary distance, and the topology was evaluated by bootstrap analysis (1,000 replicates) using the neighbor joining method.

All amplifications were performed by nested-PCR. HBV DNA extraction, preparation of PCR cocktails, and addition of templates were performed in separate areas, and negative controls in duplicate were included in each PCR reaction.

### Preparation of Peripheral Blood Mononuclear Cells

Peripheral blood mononuclear cells (PBMCs) were freshly isolated from heparinized blood by Ficoll-Hypaque density gradient centrifugation with Lymphoprep (Axis-Shield, Oslo, Norway) as previously described [7]. Subsequently, the cells were resuspended in complete RPMI-1640 medium (Gibco^®^, Invitrogen, Beijing, P.R. China), which contained 10% heat-inactivated fetal bovine serum (Gibco^®^, Invitrogen, Australia), 2 mM L-glutamine, 100 U/mL penicillin and 100 μg/mL streptomycin. After isolation, PBMCs were cryopreserved in a medium containing 90% fetal bovine serum and 10% dimethyl sulfoxide (Sigma-Aldrich, St. Louis, MO), and then stored in liquid nitrogen.

### Synthetic Peptides and Antibodies

A panel of 56 18-mer peptides overlapping by 10 residues and covering the full S and C open reading frames of the HBV was obtained from Sigma-Aldrich. These peptides were dissolved in dimethyl sulfoxide (analytic reagent obtained from Sigma-Aldrich) and were pooled in 2 mixtures, named the S peptide pool (30 peptides covering the whole HBV S-ORF) and the C peptide pool (26 peptides representing HBV C-ORF). The purity of these peptides was more than 90%. Recombinant HBsAg and HBcAg were purchased from American Research Products Inc (Belmont, MA, USA).

Peridinin-chlorophyll-protein (PerCP) labeled anti-CD8 antibody (SK1, Mouse IgG1), allophycocyanin (APC) labeled anti-CD3 antibody (UCTH1, Mouse IgG1), fluorescein isothiocyanate (FITC) labeled anti-IL2 antibody (5344.111, Mouse IgG1), phycoerthrin (PE) labeled anti-IFN-γ (25723.11, Mouse IgG2b) and their isotypes (FITC labeled Mouse IgG1 and PE labeled Mouse IgG2b) were obtained from BD Bioscience (San Jose, CA). Purified anti-CD28 monoclonal antibody was obtained from BD Pharmingen (San Diego, CA) for use in the intracellular cytokine staining assays.

### Ex vivo ELISPOT Assay for Interferon-gamma

The antigens for the human IFN-γ ELISPOT assays [8] were the 2 pools of 18-mer peptides, the rHBsAg and the rHBcAg as described above. Multiscreen-IP 96-well plates (Millipore, Billerica, MA) were coated overnight at 4°C with 10 μg/mL anti-human IFN-γ monoclonal antibody (1-DIK; Mabtech, Sweden). Plates were then washed seven times with DPBS, and blocked with RPMI 1640 supplemented with 10% fetal bovine serum for 2 hours at room temperature. Freshly isolated or frozen-thawed PBMCs (2.5 × 10^5^/well) were seeded in duplicate for either each individual peptide mixture (2 μg/mL per peptide) or each recombinant HBV antigen (10 μg/mL). Plates were incubated for 32 hours at 37°C with 5% CO2. After washing, 50 μL of 1 μg/mL biotinylated monoclonal antibody (7-B6-1; Mabtech, Sweden) was added to each well. After 2 hours of incubation at room temperature, plates were washed seven times; 50 μl per well of 1 μg/mL Streptavidin-Alkaline phosphatase (Mabtech, Sweden) was added, and the plates were incubated for a further 1 hour at room temperature. Plates were then washed seven times, and 100 μL per well of BCIP/NBT (diluted by distilled water; Zymed^® ^BCIP/NBT SUBSTRATE KIT, Invitrogen, Camarillo, CA) was added. After 10 minutes, the colorimetric reaction was stopped by distilled water and, washed three times with distilled water. Plates were air dried, and the ImmunoSpot^® ^S4 Macro Analyzer (Cellular Technology Ltd, USA) was used for spot counting. Results were expressed as numbers of spot-forming cells (SFC) per 10^6 ^PBMCs. The number of specific IFN-γ-secreting cells was calculated by subtracting the value of the unstimulated control from the value of the stimulated sample. The positive control consisted of PBMCs stimulated with phytohemagglutinin (PHA, 10 μg/mL; from Sigma-Aldrich). The criteria for a positive response for the ex vivo ELISPOT assays was more than 5 SFC per well and more than twice the number of SFC than the unstimulated control wells [9,10]. Therefore, in this study, more than 20 SFC per 10^6 ^PBMCs would be a positive response.

### Intracellular Cytokine Staining for Interferon-γ and Interleukin 2 *in vitro*

Briefly, freshly isolated or frozen-thawed PBMCs were seeded into 96-well round bottom culture plates (1 million PBMCs per well). The 2 pools of 18-mer overlapping peptides covering the S and C open reading frames of the HBV genome were used as specific stimuli. Phorbol 12-myristate 13 acetate (PMA, 50 ng/ml; Merck, Darmstadt, Germany) and ionomycin (1 μmol/L; Merck, Darmstadt, Germany) were used as positive controls. Anti-CD28 antibody was added to each well for amplification. After 2 hours of incubation at 37°C in 5% CO_2_, the transport inhibitor BD GolgiPlug™ (containing Brefeldin A; BD Pharmingen, San Diego, CA) was added for an additional 4 hours of incubation. At the end of the culture, the cells were treated with 25 mM EDTA at 37°C for 15 minutes, and then harvested. Subsequently, the cells were stained with APC labeled anti-CD3 antibody and PerCP labeled anti-CD8 antibody for 30 minutes at room temperature in darkness, and then fixed by Medium A (Caltag™, Fix&Perm^® ^reagents, Invitrogen) for 15 minutes at room temperature. Cells were then permeabilized by Medium B (Caltag™, Fix&Perm^® ^reagents, Invitrogen) in the presence of FITC labeled anti-IL2 antibody and PE labeled anti-IFN-γ or the respective isotype control antibody at room temperature for 25 minutes. Finally, the cells were washed by DPBS, containing 1% bovine serum albumin and 0.1% sodium azide, and resuspended with 1% paraformaldehyde. Two hundred thousand (2 × 10^5^) cells were acquired by BD FACSCanto™ II (BD Bioscience, San Jose, CA) and analysed with BD FACSDiva software (BD Bioscience, San Jose, CA). A positive response was considered to be 0.05% cytokine+, CD4+/CD8+T cells above the background response (response to medium and co-stimulatory molecular stimulation) in this investigation [11].

### Statistical Analysis

Statistical analysis was performed using the Mann-Whitney U test or Kruskal-Wallis H test for comparison of continuous variables between study groups. The rates of positive response were compared by Fisher's exact test. The SPSS statistical package version 15.0 (SPSS Inc., Chicago, IL, USA) was used for analysis while statistical significance was assessed at the 0.05 level (*P *< 0.05).

## Results

### The Demographic and Serological Characteristics of Patients with HBsAg Seroclearance

The age, gender, HBV DNA levels and ALT levels in all subjects are shown in Table 2. The patients with HBsAg seroclearance showed a variety of patterns of serological HBV markers, but they were all positive for anti-HBc antibody. Eight patients were anti-HBs positive, and 2 of these were seropositive for HBeAg at the time we collected the blood sample. However, both subjects became HBeAg-negative during follow-up (Table 1).

### Virological Analysis in Patients with HBsAg Seroclearance

A 583 bp fragment from the HBV S open reading frame was amplified and sequenced from 6 of the 14 subjects with HBsAg seroclearance. The HBV genotypes and the repertoire of amino acid mutations in the reverse transcriptase region of the P and overlapping S polypeptides were identified by aligning the sequences with 242 genotype C sequences from GenBank. Five of the 6 sequences were genotype C and one was genotype B. We found that two lamivudine-treated patients infected with a genotype C HBV developed L80I+M204I+L180M triploid mutants in the reverse transcriptase (RT) region. The W196L mutation in the S region in these two patients corresponded to M204I in the RT region (Table 1). The other variants identified in the RT region and S regions (Table 1), have not been previously reported in occult HBV infection, and no evidence have proved that they would be attributed to the loss of HBsAg in vitro or in vivo.

### The Production of Interferon-gamma in HBV-Specific T Cells through Ex Vivo ELISPOT Assay

**Figure 1:**
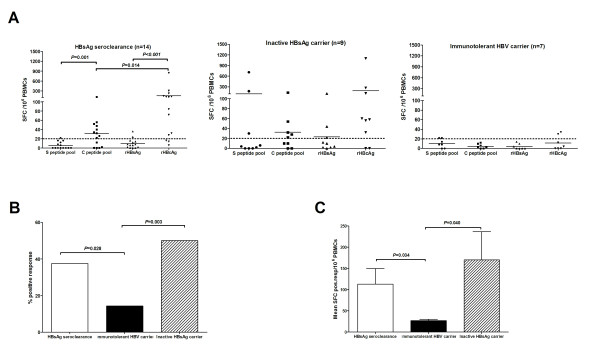
**Ex vivo IFN-γ ELISPOT responses to HBV S and C open reading frame peptide pools and recombinant HBV proteins**. (A) Comparison of subjects with HBsAg seroclearance, immunotolerant HBV carriers and inactive HBsAg carriers. Each line represents the mean number of spot-forming cells induced by each stimulus. Each dotted line represents the cut-off value for a positive test in the ELISPOT assay. (B) Comparison of the frequency of positive responses obtained in HBsAg seroclearance and control populations. (C) Comparison of the mean number of spots/well from the subjects with positive tests in each group. Each bar represents the mean ± SD.

First, we wondered whether an HBV-specific T-cell response is detectable in patients with HBsAg seroclearance. In addition, we asked about the antigen specificity and the magnitude of the detected responses. Both the peptide pools and both the recombinant HBV proteins were used to stimulate PBMCs in an *ex vivo *ELISPOT assay for IFN-γ. HBV-specific T cells could be detected in patients with HBsAg seroclearance at a frequency ranging between 22 and 846 antigen-specific T cells per 10^6 ^PBMCs (Figure 1A). The immune responses in patients with HBsAg seroclearance were mainly to the C peptide pool and the rHBcAg (9 of 14 and 11 of 14 positive tests respectively). There were only three positive responses to S open reading frame antigens, and these were all close to the baseline response (Figure 1A). Consistent with this, patients with HBsAg seroclearance displayed a more robust T-cell response against rHBcAg than against rHBsAg (Figure 1A, *P *< 0.001), and against the C peptide pool relative to the S peptide pool (Figure 1A, *P *= 0.001). Furthermore, rHBcAg could prime a more dramatic HBV-specific T-cell response than the C peptide pool in patients with HBsAg seroclearance (Figure 1A, Mean ± SD, 152.1 ± 231.9 VS. 47.1 ± 27.6 SFC pos resp/10^6 ^PBMCs, Mann-Whitney U test, *P *= 0.014).

The frequency of positive responses in patients with HBsAg seroclearance was higher than that in immunotolerant HBV carriers (37.5% VS. 14.3%, *χ*^2 ^= 4.812, *P *= 0.028, Figure 1B). The frequency of positive responses in the inactive carriers was also higher than that in the immunotolerant HBV carriers (50.0% VS. 14.3%, *χ*^2 ^= 8.905, *P *= 0.003, Figure 1B). The mean values of spot-forming cells in positive responses from subjects with HBsAg seroclearance were similar to those from the inactive HBsAg carriers (Mean ± SD, 113.0 ± 174.6 VS. 169.9 ± 282.7 SFC pos resp/10^6 ^PBMCs, Mann-Whitney U test, *P *= 0.723). They were both more vigorous than those from immunotolerant HBV carriers (Figure 1C, Mean ± SD, 113.0 ± 174.6 VS. 27 ± 6 SFC pos resp/10^6 ^PBMCs, *P *= 0.034 and 169.9 ± 282.7 VS. 27 ± 6 SFC pos resp/10^6 ^PBMCs, *P *= 0.040; by Mann-Whitney U test, respectively).

### The Response of Interferon-gamma in HBV-Specific T Cells Determined by Intracellular Cytokine Staining *in vitro*

The purpose of the intracellular cytokine staining assays was to determine the phenotype of the HBV-antigen specific, interferon-gamma producing T cells found in the ELISPOT assay. Both CD8+ (Figure 1A) and CD4+ (Figure 1B) peptide-specific T cells were identified in all 3 groups of subjects. In the HBsAg seroclearance group, the frequency of both CD8+ (*P *= 0.040) and CD4+ (*P *= 0.033) T cells responding to the C peptide pool was higher than the frequency of cells responding to the S peptide pool. This is consistent with the results of the ELISPOT assay.

**Figure 2:**
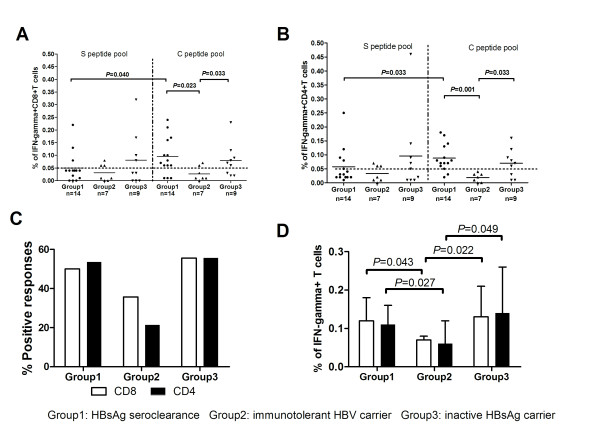
**Results of the intracellular cytokine staining assay for interferon gamma**. (A) Individual responses of CD8+ T cells stimulated by HBV peptide pools in subjects with HBsAg seroclearance (Group1), immunotolerant HBV carriers (Group2) and inactive HBsAg carriers (Group3). (B) Individual responses of CD4+ T cells stimulated by HBV peptide pools in subjects with HBsAg seroclearance, immunotolerant HBV carriers and inactive HBsAg carriers. (C) The mean frequencies of positive CD4+ and CD8+ T cell responses in the three populations. (D) Comparison of mean frequencies of IFN-g+ secreting CD8+ and CD4+ T cells in subjects with positive responses to either of the peptide mixtures in the three groups. Each dotted line in 2A and 2B represents the cut-off value for a positive test. Each bar in 2D represents the mean ± SD percentage of IFN-g secreting CD4+ and CD8+ cells in subjects with positive responses to either the C or S peptide pools.

The percentage of IFN-γ producing CD8+ and CD4+ T cells induced by the C peptide pool in both the HBsAg seroclearance and inactive HBsAg carrier groups was higher than that in the immunotolerant HBV carriers (Figures 2A and 2B). There was also a higher frequency of positive responses to the C peptide pool in both the HBsAg seroclearance and inactive carrier groups than in the immunotolerant group (Figure 2C), but these differences did not reach statistical significance. However the frequencies of interferon-gamma producing CD4+ and CD8+ T cells in the positively-responding subjects was higher in both the HBsAg seroclearance and inactive carrier groups than in the immunotolerant group (Figure 2D).

### The Production of Interleukin 2 in HBV-Specific T Cells Determined by *in vitro *Intracellular Cytokine Staining

**Figure 3:**
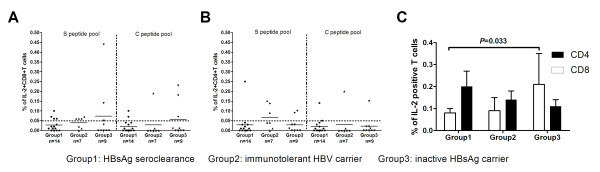
**Results of the intracellular cytokine staining assay for interleukin 2**. (A) Individual responses of CD8+ T cells stimulated by HBV peptide pools in subjects with HBsAg seroclearance (Group1), immunotolerant HBV carriers (Group2) and inactive HBsAg carriers (Group3). (B) Individual responses of CD4+ T cells stimulated by HBV peptide pools in subjects with HBsAg seroclearance, immunotolerant HBV carriers and inactive HBsAg carriers. (C) The mean frequencies of positive CD4+ and CD8+ T cell responses in the three populations. Each bar in 3C represents the mean ± SD percentage of IL-2 secreting CD4+ and CD8+ cells in subjects with positive responses to either the C or S peptide pools.

Interleukin 2 (IL2) is an alternative index of cell proliferation and activation capacity. Therefore, in order to assess the proliferative response to HBV antigen in vitro, we measured the IL2 responses to the C and S peptide pools in both CD4+ and CD8+ T cells in the three groups of subjects. The individual responses of CD8+ T cells to the C and S peptide pools are shown in Figure 3A and the individual responses of the CD4+ T cells are shown in Figure 3B. IL2 was produced by CD4+ and CD8+ T cells in some subjects from all 3 subject groups, in response to both C and S peptide pools. There were no differences between the frequency or magnitude of the IL2 responses to the C and S peptide pools. There was no difference in the frequency or magnitude of these responses among the three subject groups.

## Discussion

HBsAg seroclearance following nucleos(t)ide analogue or pegylated interferon α treatment is a rare event in chronic hepatitis B. Since HBsAg seroclearance may result in a decreased risk for hepatocellular carcinoma in these patients [12], it will be worthwhile identifying any immune mechanisms that might contribute to HBsAg seroclearance, as they may provide targets for immunotherapy. The mechanisms that might contribute include acquired CD4+ and CD8+ T cell responses [13], innate immune responses [14], and mutations in the HBsAg arising from genetic drift [15]. The possibility that HBsAg seroclearance results from an acquired immune response to antigens within the envelope proteins is of particular interest.

In this study, we have focused on the frequency and magnitude of acquired CD4+ and CD8+ T cell responses to both the HBV core and envelope proteins. Our data are consistent with previously published data[16,17], in that they show both a higher frequency (Figures 1B and 2C) and magnitude (Figures 1C and 2C) of antigen-specific, CD4+ and CD8+ T cell responses to core and envelope antigens in subjects with inactive HBeAg-negative chronic hepatitis B virus infection relative to subjects who are immunotolerant. This was found with both ELISPOT and intracellular cytokine staining assays for interferon gamma. The frequency and magnitude of antigen-specific T cell responses was also higher in the HBsAg seroclearance group than in subjects with immunotolerance. However the T cell responses in the seroclearance group were almost exclusively to core antigens. Only a small number of low level responses to envelope gene antigens were detected. This is an important negative result for two reasons. It suggests that acquired T cell responses to envelope antigens do not contribute to HBsAg seroclearance following anti-viral therapy in Chinese subjects. In addition, it does not support the possibility that CD8+ T cell exhaustion can be reversed by a reduction of antigen levels in vivo. One possible, but unlikely, limitation to these conclusions is that this HBsAg seroclearance group was comprised of subjects with a biased repertoire of class I and class II alleles that do not present envelope peptides effectively. It is also possible that a longer period of antigen loss would be needed to allow these T cells to recover.

The additional finding that there was no increase in the responses to core antigens beyond that detected in inactive carriers suggests that neither acquired immune response drives HBsAg seroclearance in the post-treatment situation. However a comparison of anti-core responses in HBeAg-negative, treated subjects who did and did not undergo HBsAg seroclearance would be necessary to fully test this hypothesis.

Thus our data did not provided clues to the cause of HBsAg seroclearance in this group of subjects. The possibility that HBsAg seroclearance occurs as a result of mutations accumulating in the envelope protein as a result of genetic drift has not been excluded by direct sequencing of PCR products. There may be a large number of harmful mutations in the envelope gene that each occurs at low frequency in the viral population. Phylogenetic analysis of cloned envelope gene sequences would be necessary to exclude this possibility. The possibility that the innate immune system is responsible for HBsAg seroclearance is currently difficult to test, as the mechanisms by which the HBV might potentially be controlled by innate immunity are unknown.

The failure to find evidence that acquired immunity might drive HBsAg seroclearance in these subjects does not exclude the possibility that immunotherapy directed at envelope peptides may eventually have a role in improving responses to antiviral therapy. The finding that it is possible to reverse the exhaustion of CD8+ T cells that respond to envelope antigens in vitro [5] suggests that potentially useful CD8+ T cells exist in the blood of these patients. Further research into how they might be activated in vivo is still needed.

## Conclusions

According to our data, we have been unable to detect an increase in CD4+ or CD8+ T cell immunity to HBV envelope proteins in Chinese subjects who have undergone HBsAg seroclearance following antiviral therapy. This suggests that acquired immunity to HBV envelope antigens is not an important cause of HBsAg seroclearance in these patients. In addition, this study does not support the hypothesis that a sustained decrease in the level of the HBV envelope protein in vivo will result in a reversal of exhaustion of the CD8+ T cells that respond to envelope peptides.

## Abbreviations

DPBS: Dulbecco's phosphatase buffer saline; ELISPOT: enzyme-linked immunosorbent spot; HBcAg: hepatitis B core antigen; HBeAg: hepatitis B e antigen; HBsAg: hepatitis B surface antigen; HBV: hepatitis B virus; IFN-γ: interferon-gamma; IL2: interleukin 2; ORF: open reading frame; PCR: polymerase chain reaction; rHBcAg: recombinant HBcAg; rHBsAg: recombinant HBsAg; SFC: spot-forming cell.

## Competing interests

The authors declare that they have no competing interests.

## Authors' contributions

MFL carried out the Elispot assays, participated in the design of study and drafted the manuscript. SWM participated in the design of study, and carried out the collection of Elispot data. XXH participated in the Elispot and ICS assays and helped to perform the statistical analysis. BZ participated in the sequence alignment and helped to draft the manuscript. JCZ participated in the collection of clinical data and samples. JJC participated in the design of the study and helped to perform the statistical analysis. ZHW participated in the sequence alignment and helped to draft the manuscript. JS participated in the design of the study and helped to draft the manuscript. XL Z participated in the samples' collection and a part of immunoassays. WA conceived of the study and helped to draft the manuscript. JLH obtained the funding, conceived of the study, and participated in its design and coordination and helped to draft the manuscript. All authors read and approved the final manuscript.
